# A Hidden Markov Ensemble Algorithm Design for Time Series Analysis

**DOI:** 10.3390/s22082950

**Published:** 2022-04-12

**Authors:** Ting Lin, Miao Wang, Min Yang, Xu Yang

**Affiliations:** School of Computer Science and Technology, Beijing Institute of Technology, Beijing 100081, China; linting@bit.edu.cn (T.L.); 3220201093@bit.edu.cn (M.W.); 3220180890@bit.edu.cn (M.Y.)

**Keywords:** time series analysis, ensemble learning, Wasserstein distance, hidden Markov model, conditional variance autoencoder

## Abstract

With the exponential growth of data, solving classification or regression tasks by mining time series data has become a research hotspot. Commonly used methods include machine learning, artificial neural networks, and so on. However, these methods only extract the continuous or discrete features of sequences, which have the drawbacks of low information utilization, poor robustness, and computational complexity. To solve these problems, this paper innovatively uses Wasserstein distance instead of Kullback–Leibler divergence and uses it to construct an autoencoder to learn discrete features of time series. Then, a hidden Markov model is used to learn the continuous features of the sequence. Finally, stacking is used to ensemble the two models to obtain the final model. This paper experimentally verifies that the ensemble model has lower computational complexity and is close to state-of-the-art classification accuracy.

## 1. Introduction

Time series data analysis [1] is often used to solve two types of tasks, one is regression tasks, which generate new series based on existing data, and the other is classification tasks, which determine a given series class [2]. In this paper, we focus on processing time series similarity to solve the classification task [3,4]. Classical classification methods include the following three. First, methods based on distance metrics. Second, methods based on the distribution metrics. Third, neural network-based methods. Although the above methods have achieved some success, they all have shortcomings. For example, sensitivity to noise, strict data normality requirements, and long training time. We believe that the main reason for these problems is the low utilization of time series data information. Time series data features include discrete features and continuous features. Learning both types of features in one model naturally improves the efficiency of information utilization. Based on this, we design an ensemble model. The model has a low computational complexity while maximizing information mining.

As mentioned earlier, time series data features have two properties. We first address the learning of continuous features. The features are implicitly represented in the data. Therefore, we use hidden Markov model (HMM) [5] to learn the continuum features of the series. HMM is a traditional time series analysis model based on likelihood probabilities that can mine hidden state sequences.

To obtain the dispersion characteristics of a time series, we consider using conditional variance autoencoder (CVAE) [6]. CVAE is a method for learning data distribution, but it has drawbacks. CVAE uses Kullback–Leibler Divergence (KL Divergence) [7] to measure the distance of a distribution. However, KL Divergence has two drawbacks. 1. KL Divergence does not conform to symmetry, so it is not a measurement function. 2. KL Divergence cannot measure two distributions that do not have overlapping parts. As time series data can be viewed as a high-dimensional information space mapped in a low-dimensional manifold, the probability of their support set overlapping part measure being 0 approaches 1 [8]. Based on this, we choose to use Wassersteins distance [9] to measure the distance of the time series. Therefore, we designed conditional variance autoencoder based on Wassersteins distance (CVAEWD).

Having solved the problem of how to obtain sequence features of different nature, the challenge is to ensemble and utilize these features effectively. Through the analysis, we choose to ensemble HMM and CVAEWD with stacking [10] to obtain the HMM-CVAEWD ensemble model (HCW), which ensures the full utilization of the data. We also design experiments to compare HCW with some advanced methods. The following are the main contributions of this paper.

(1) Wassersteins distance is introduced into CVAE as a time series difference measurement function, which makes the judgment of different series differences more accurate;

(2) Find a suitable ensemble learning algorithm through experiments, and merge the data information (continuity) and distribution information (discreteness) of the time series to obtain the HCW ensemble model;

(3) Comparative experiments prove that the ensemble algorithm proposed in this paper has higher accuracy and learning efficiency.

The paper is organized as follows: Section 2 presents the related work. Section 3 focuses on the construction process of the HMM classifier and the CVAEWD classifier. Section 4 proposes the method to optimize HCW; Section 5 shows the experimental framework and experimental results. The last section covers the analysis and research conclusions.

## 2. Related Work

This paper attempts to solve the classification problem of time series analysis. The approach to solving this problem is roughly two steps, which are (1) choosing a suitable metric. (2) mining the hidden information of the sequences. The first step can start with local metric and global metric.

The idea of the distance-based approach is straightforward: as long as we find a way to measure the distance of different sequences [11], we can determine the similarity of the sequences. Dynamic Time Warping (DTW) [12] is the most classical distance metric, which can be used to locally scale the sequences on the time axis, which overcomes the problem that unequal sequences cannot be matched one by one [13]. However, the drawbacks of DTW are obvious: it is sensitive to noise and has high computational complexity. To solve the drawbacks of DTW, String-Edit distance [14] is proposed. The well-known algorithm is Longest Common Subsequence (LCSS) [15,16], which is more adaptable to different data in a short time, such as breakpoints, and thus has stronger noise immunity. However, LCSS cannot solve displacement noise. Based on this, Edit Distance on Real Sequence (EDR) was born [17], and EDR has stronger robustness than LCSS. In summary, the advantages and disadvantages of distance metrics are obvious. For today’s time series data, the longer data length and greater complexity make the computational complexity of these methods steeper. Therefore, better methods need to be explored.

The distance metric of a time series can be seen as a measure of local variability, and then the distribution metric is a measure of overall variability. The most commonly used method is Kullback–Leibler Divergence (KL Divergence). Attias et al. started KL Divergence as a similarity metric function in 2000 [18]. The objective function of the variational autoencoder (VAE) proposed by Diederik et al. in 2013 consists of KL Divergence [19]. In 2018, Shuai Wang et al. improved the KL Divergence to make it more suitable as the loss function of neural networks [20]. Although KL Divergence has a wide range of applications, KL Divergence is not concerned with the geometric properties of the sample space and is not computationally generalizable to all types of sample spaces. Therefore, another distribution metric, Optimal Transport (OT), started to develop. The one that is now widely used is Wasserstein distance. In 2017, Arjovsky et al. used Wasserstein distance (W-Distance) [21] to construct Generative Adversarial Network (GAN) to obtain better stability. In the same year, Eric Xing et al. linked GAN and VAE to explore the discrepancy between KL Divergence and W-Distance [22].

After selecting the appropriate measurement function, the maximum utilization of hidden information of the time series has become another research hotspot. In 2000, Oates et al. combined DTW and HMM. Subsequently, more research focused on using machine learning or neural network methods to mine hidden information. In 2015, Lv et al. applied Artificial Neural Network (ANN) to time series mining [23], and established a prediction model based on intelligent theory [24]. In 2021, Sosiawan et al. combined Genetic Algorithm and HMM to solve the problem of time series data mining [25]. In the same year, Ilhan et al. used Recurrent neural network (RNN) [26] and HMM to build an adaptive time series forecasting model [27]. Therefore, this paper uses HMM to solve the hidden information mining problem.

## 3. Time Series Data Classification Algorithm Based on HCW

**Figure 1:**
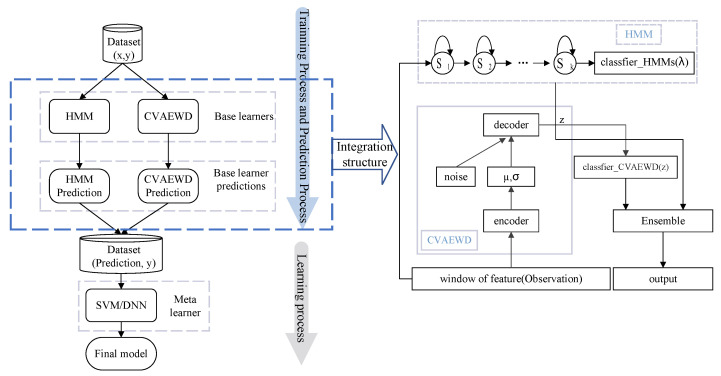
Schematic diagram of HCW overall structure and stacking structure.

According to the description in the first part, we first construct the HMM classifier and CVAEWD classifier separately, and then ensemble them with stacking to obtain the HCW ensemble model. Figure 1 shows the overall structure of the HCW model and ensemble structure.

The left half of Figure 1 shows the overall structure of the HCW model, integrating the results of two weak classifiers and sending them to the meta learner. The right half shows the ensemble structure, where the HMM classifier and the CVAEWD classifier are each classified and their results are ensemble and output. Next, the two weak classifiers and stacking method are elaborated.

### 3.1. HMM Classifier

**Figure 2:**
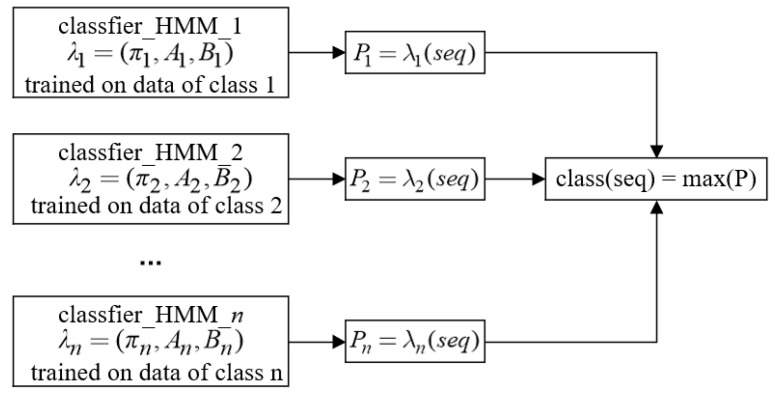
HMM classifier structure diagram.

HMM is based on the Markov model, which is used to describe a Markov process with hidden unknown parameters, and is a kind of dynamic state space model. The observation state sequence of time series data has a certain probability relationship with the hidden process, and the HMM model includes Markov process and the observable state related to the hidden state [28], as the Markov process changes with time. Therefore, the time series data and the HMM model can be adapted. The HMM model learning and training classifier and the discrimination process are shown in Figure 2.

The HMM classifier needs to train and learn *n* classifiers, $\lambda_{1},\lambda_{2},\cdots ,\lambda_{n}$, for each type of data. Next, we input the time series into the classifiers of all categories and get the corresponding probabilities $p_{1},p_{2},\cdots ,p_{n}$. For a single HMM model, we take the category label of the maximum value of all results as the final classification result.

### 3.2. CVAEWD Classifier

CVAEWD is derived from Variational Autoencoder (VAE) [29], so it has some of the characteristics of VAE, including stable learning and training, encoder-decoder architecture mode, good latent manifold structure, etc. [30].

#### 3.2.1. Comparison of KL Divergence and W-Distance

**Figure 3:**
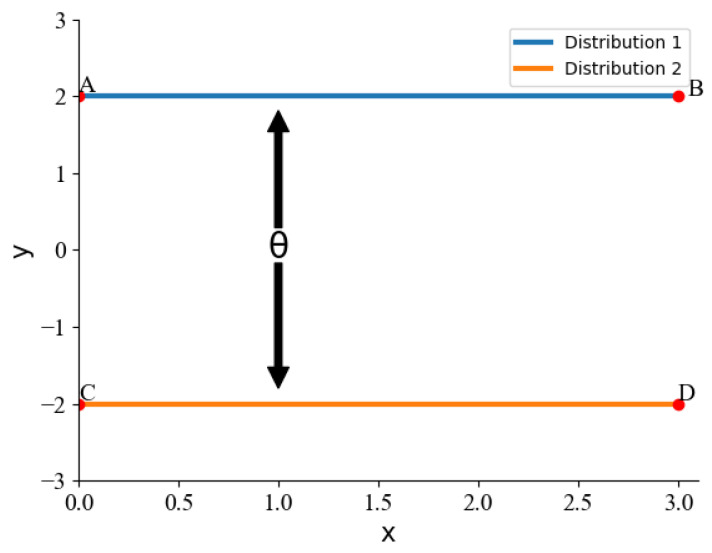
Schematic diagram of the two different distributions.

When two distributions have no overlapping parts, KL Divergence cannot be measured, while W-Distance can give continuous values. As shown in Figure 3, we consider two distributions $p_{1}$ and $p_{2}$ in two dimensions, with $p_{1}$ uniformly distributed on line AB and $p_{2}$ uniformly distributed on line CD, and control the distance between the two distributions through the θ. KL Divergence is calculated as Equation (1), and the W distance is calculated as Equation (2)

(1) $$D_{KL}\left(p\left(x\right)||q\left(x\right)\right)=\sum_{x}p\left(x\right)log\frac{p(x)}{q(x)}$$

(2) $$W\left[p\left(x\right),q\left(x\right)\right]=\underset{\gamma \in \Gamma (p(x),q(x))}{inf}\;E_{\left(x,y\right)∼\gamma }\left[c\left(x,y\right)\right]$$

(3) $$\left\{\begin{array}{c} DKL\left(p1||p2\right)=\left\{\begin{array}{cc} +\infty & \theta \neq 0\\ 0 & \theta =0 \end{array}\right.\\ W(p1,p2)=|\theta | \end{array}\right.$$

where p(x) and q(x) denote the expressions of the two distributions. $\left(x,y\right)∼\gamma$ is any binary distribution about with marginal distributions p(x) and q(x). c(x,y) is the cost function, and for any *x* value under the *p* and any *y* value under the *q*, $c\left(x,y\right)\geq 0$ and $c\left(x,x\right)=0$ are guaranteed. Bringing the two distributions into the calculation, the final result is obtained as Equation (3).

From the calculation results, it can be seen that if the two distributions do not intersect, and if KL Divergence is used as the Loss function of CVAEWD, the algorithm will not converge because there is no gradient. In contrast, W-Distance is a continuous value and can be learned. Therefore, to improve the robustness of the ensemble model, we choose W distance instead of KL Divergence.

#### 3.2.2. Structure of CVAEWD

**Figure 4:**
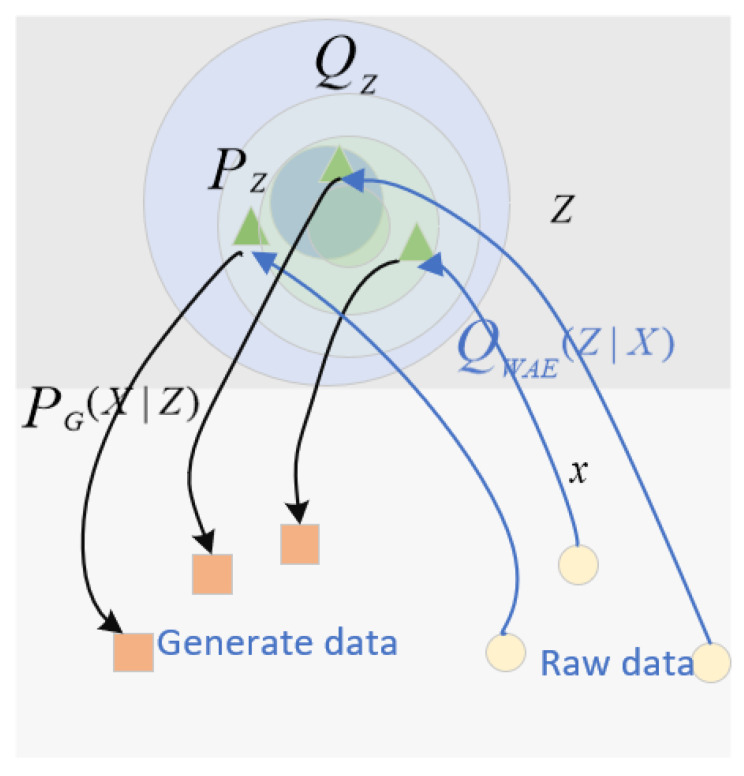
Reconstruction diagram of CVAEWD.

CVAEWD is a variation from Variational Autoencoder (VAE). VAE provides a probabilistic way of describing hidden space observations. Therefore, an encoder is constructed to describe the probability distribution of each hidden attribute. CVAEWD replaces the KL Divergence in loss function of VAE with the W-Distance. In addition to that, the encoder needs to ensure that sufficient information in the hidden variables is maintained in the learned training samples for reconstruction. The reconstruction structure of CVAEWD is shown in Figure 4.

As can be seen from the figure, the reconstruction process is such that the expectation $Q_{z}$ of the hidden variable *z* under the $p_{x}$ distribution goes to match the prior $P_{z}$ to get $P_{G}\left(X|Z\right)$, which allows different samples to keep distance from other samples. Also, CVAEWD does not need to construct a distribution for each data due to the advantage of W-Distance and can use deterministic encoders. In contrast, VAE can only use Gaussian encoders.

Since this paper aims to solve the classification problem using sequence similarity, the CVAEWD generation model is to be transformed into a basic classifier model. The generative model constructs the data *x* from the label *y*, and the classification model obtains the label *y* from the data *x*. Therefore, the data *x* and label *y* in the CVAEWD formula can be swapped, and the likelihood function is obtained as Equation (4).

(4) $$\begin{array}{cc} \log p_{\theta }\left(y|x\right) & =dis_{wa}\left(q_{\phi }\left(z|x,y\right)\|p_{\theta }\left(z|x,y\right)\right)\\ \; & +\Gamma (\theta ,\phi ;x,y) \end{array}$$

Among them, θ and ϕ denote the generated data and labels. $dis_{wa}$ denotes W-Distance of the generated data and raw data. The definition of Γ(θ,ϕ;x,y) is expressed as Equation (5):

(5) $$\begin{array}{cc} \Gamma (\theta ,\phi ;x,y) & =-dis_{wa}\left(q_{\phi }\left(z|x,y\right)\|p_{\theta }\left(z\right)\right)\\ \; & +E_{q_{\phi }\left(z|x,y\right)}\log p_{\theta (x|y,z)} \end{array}$$

We reparameterize $q_{\phi }\left(z|x,y\right)$ as $z=g_{\phi }\left(x,y,\varepsilon \right),\epsilon ∼N\left(0,1\right)$, and set an appropriate distribution in the model. After the learning and training are completed, the model can be used as a classifier to predict the label of the input *x*. The prediction process can be expressed as Equation (6). At this point, the CVAEWD classifier has been constructed.

(6) $$y^{*}=\arg\max p_{\theta }\left(y|x,z^{*}\right),z^{*}=E\left[z|x\right]$$

### 3.3. Ensemble Strategy

The reasons why ensemble learning is effective are discussed in terms of statistics, computation, and representation, respectively. Statistically speaking, a learning algorithm can be understood as finding the best hypothesis in the hypothesis space. However, when the amount of data in the training sample is too small to be used to learn the target hypothesis accurately, the learning algorithm can find many classifiers that satisfy the training sample. Therefore, the learning algorithm faces some risk of misclassification when selecting any classifier but can reduce the risk of selecting the wrong classifier by fusing multiple hypotheses through an ensemble strategy. Computationally speaking, many learning algorithms are likely to fall into the error of local optimality when performing an optimization search, so it is not easy to obtain a globally optimal hypothesis for learning algorithms. Artificial neural networks and decision trees are an NP problem. Ensemble algorithms can perform local searches from multiple starting points, thus reducing the risk of falling into bad local minima. In most application scenarios, no hypothesis in the hypothesis space can represent (or approximately represent) the true classification function f. Therefore, the hypothesis space can be expanded by a weighted form for different hypothesis conditions. The learning algorithm can find an approximation to the function *f* in a hypothesis space that cannot represent or approximately represent the true classification function *f*. With the above ensemble idea, the ensemble strategy is designed based on both weighting method and learning strategy.

#### 3.3.1. Weighting Method

The first way of the weighting method is the simple averaging method, assuming that the prediction categories are $c_{1},c_{2},\cdots ,c_{n}$, and for any prediction sample *x*, the prediction results of *T* weak learners are $h_{1}\left(x\right),h_{2}\left(x\right),\cdots ,h_{n}\left(x\right)$. Then the prediction results of the *T* weak learners for sample *x* according to the category $c_{i}$ with the highest number of prediction results is the final classification category. If more than one category receives the highest votes, one is randomly selected as the final category. Based on this, the simple averaging system is based on the maximum results of the HMM classifier and the CWAE classifier, respectively, as the final decision. The mathematical expression is shown in Equation (7).

(7) $$\begin{array}{c} class_{HMM-CVAEWD}\left(seq\right)=\\ c_{argmax_{i}}\left(\sum_{i}^{M}\;\frac{class_{HMM_{i}}\left(seq\right)+class_{CVAEWD_{i}}\left(seq\right)}{2}\right) \end{array}$$

where the subscript *i* is the category and the range belongs to [1,M], and *M* is the total number of categories.

The second way is the weighted average [31], which can also be understood as a weighted voting method, that is, the result of each base learner is multiplied by the corresponding weight, and the weighted votes sum the results of all categories. The sum of the weights of the classifier should be equal to 1, and the category corresponding to the maximum value of the result is the final category. The calculation formula is shown in Equation (8).

(8) $$\begin{array}{cc} class_{HMM-CWAE}\left(seq\right) & =c_{argmax_{i}}(\underset{i}{\sum^{M}}\;\alpha_{i}class_{HMM_{i}}\left(seq\right)\\ \; & +\beta_{i}class_{CWAE_{i}}\left(seq\right)) \end{array}$$

Among them, the parameters α and β are both greater than 0 and satisfy Equation (9).

(9) $$\sum_{i}^{M}\left(\alpha_{i}+\beta_{i}\right)=1$$

The parameter values will be further determined by the neural network learning and training fitting results.

#### 3.3.2. Learning Strategy

Stacking is an ensemble learning algorithm where a meta-classifier aggregates multiple classifications. First, the base-level model is trained based on the complete learning training set, and then the meta-model is trained based on the output of the base-level model. The base-level model is usually composed of different learning algorithms, so stacking is usually heterogeneous, and the stacking algorithm is divided into two layers. The first layer is to form T weak classifiers with different algorithms, generate a new dataset of the same size as the original dataset, and then use this new dataset and fuse the weak classifiers to form the second layer of classifiers.

The second layer model needs to be further fitted to the output results of the first layer model to achieve classification. Ensemble learning itself has a certain risk of overfitting. Therefore, one direction of the second-layer classifier in this algorithm uses a simpler nonlinear model to support vector machines (SVM) [32,33]. The other direction is to use a deep neural network (DNN) [34] for optimization experiments.

In summary, we obtained four methods to ensemble the HMM classifier and CVAEWD classifier: 1. simple average ensemble method, 2. weighted average ensemble method, 3. SVM classifier ensemble method, and 4. DNN classifier ensemble method. The next chapter will find the most suitable ensemble method strategy through experiments.

## 4. Experiment and Analysis

**Figure 5:**
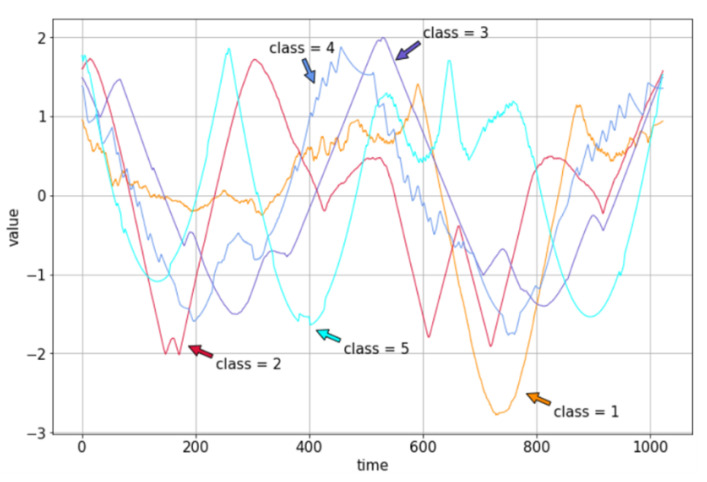
Comparison chart of different data classes.

To verify the effectiveness of the HCM learning algorithm proposed in this paper, the Mixed Shapes Small Train dataset [35] is used for learning and classification. The dataset is a time series dataset with a length of 2525 and a width of 1024. The categories are divided into five categories with different trends. The example is shown in Figure 5.

This algorithm uses predictive label accuracy and ROC curve as the model evaluation criteria. This is because it needs to be an index that can be accurately evaluated even if the sample is unbalanced. All experiments are done on a computer with a CPU model of i7-7700k, two GPU models of NVIDIA GeForce GTX 1080, 32 g RAM, and an operating system of Ubuntu 20.04.2.

The data quality can affect the quality of the learning and training results. Due to the complex network structure and a large number of parameters, to make the cohesion between the classes as high as possible, the Locality Sensitive Hash (LSH) algorithm is used to delete part of the data [36]. For five types of data, we determine the LSH similarity threshold, and calculate the result of the proportion of saved data. The threshold is shown in Table 1. Experiments were conducted using the processed data.

### 4.1. Weak Classifier Performance Experiments

**Figure 6:**
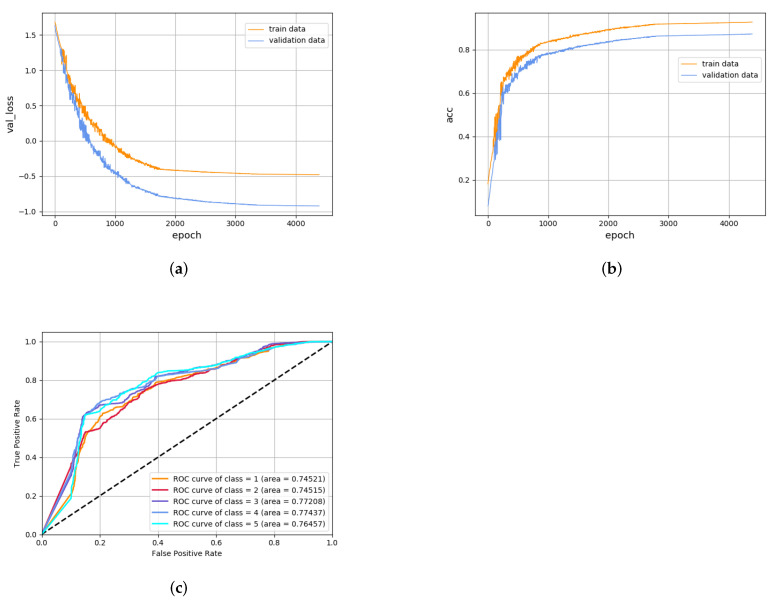
Training results of CVAEWD classifier and HMM classifier. (**a**) The loss trend graph of the CVAEWD model in the training set and the test set. (**b**) The accuracy trend graph of the CVAEWD model on the training set and the validation set. (**c**) HMM model ROC curve.

The preliminary training aims to train the model in the optimal state of the HMM classifier and CVAEWD classifier. The results are shown in Figure 6. Figure 6a plots the Loss decline curves during the training of CVAEWD, and two curves in each of the two plots are shown as labeled: the results on the training set and the test set, respectively. Figure 6b shows the accuracy curves of the CVAEWD model, and Figure 6c shows the ROC curves of the HMM model. For comparison, the five categories are drawn under the same coordinate system.

From Figure 6, it can be seen that the model performs significantly better than the test set on the training set. The CVAEWD classification model has a prediction accuracy of 83.36% with a high loss value and the HMM model has a prediction accuracy of 82.82%. Further observation can be judged, as the model appears to show the overfitting phenomenon. From the AUC curve, it can be seen that the model has deviations in the judgment of the classified data, and the difference in the AUC difference between the best classification result and the worst classification result is 0.0228. This indicates that the classification performance of the weak classifier alone is not high, and it needs to be used after integrating two models.

### 4.2. Ensemble Strategy Experiments

**Figure 7:**
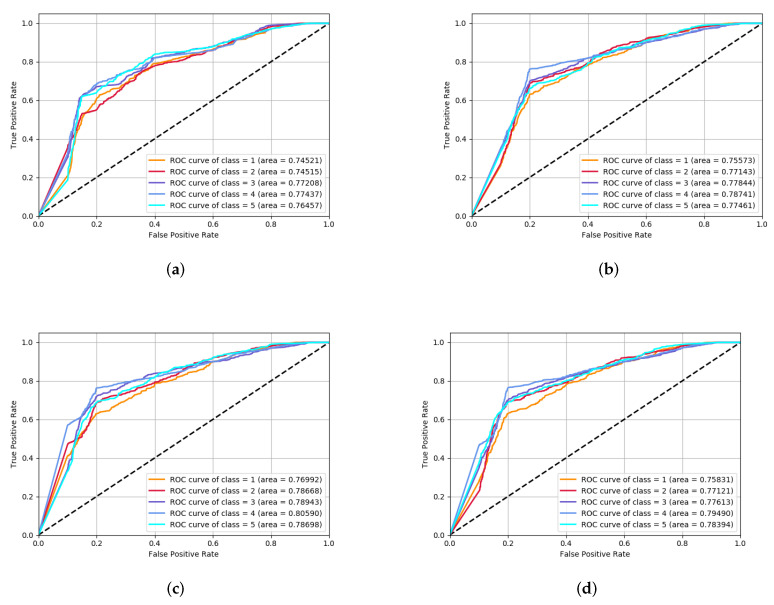
Training results of the CVAEWD and HMM classifier. (**a**) ROC curve of the simple average. (**b**) ROC curve of the weighted average. (**c**) ROC curve of SVM. (**d**) ROC curve of DNN.

According to the optimization scheme proposed in Section 3, the experiments were carried out respectively, and the ROC curve was used to evaluate the model. Among them, the fusion method of the weighting method adopts the strategy of 0.1, 0.01, and 0.001 as the interval, greedy parameter adjustment, and finally for the five types of data, the optimal weight results obtained are shown in Table 2:

After going through the process of adjusting parameters including HMM, CVAEWD, SVM, and DNN, the ROC curve of the model prediction results under the final four fusion schemes is shown in Figure 7.

Figure 7a,b are the simple average and weighted average of weight methods. Figure 7c,d are the SVM classifier and the DNN classifier of the second-layer learner in learning strategy. As can be seen from Figure 7, the sensitivity of different fusion strategies to the classification of categories is the same, and the hierarchical distinction between categories is slightly different. Therefore, based on the results of five classifications, the second-level classifier selected as the SVM classification model has the best effect on the final classification.

### 4.3. Optimization Experiments

The experiments in Section 4.1 reveal that the weak classifier appears to be overfitted. According to the experiments in Section 4.2, the SVM learning strategy is chosen for the ensemble strategy, and the SVM method is also prone to overfitting. Therefore, some methods must be used to stop model overfitting. This paper conducts experiments on the lateralization and dropout layers [37], respectively, to find the most suitable optimization method. The structure and parameters of CVAEWD after adding the dropout layer are shown in Table 3.

**Figure 8:**
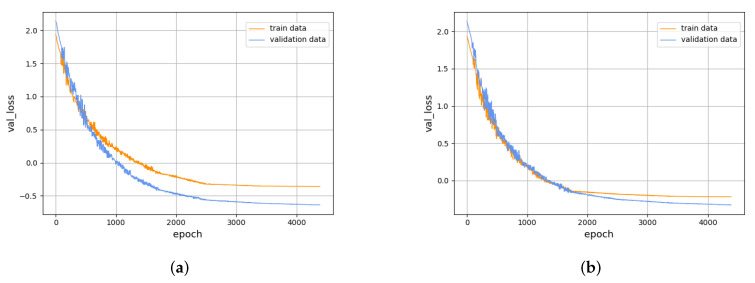
Training results of the CVAEWD and HMM classifier. (**a**) Loss curve after adding the regularization penalty term. (**b**) Loss curve after adding the dropout layer.

The results of the regularization [38] penalty term and dropout layer experiments are shown in Figure 8. In Figure 8, Figure 8a is the effect picture after adding the regularization penalty item, and Figure 8b is the effect picture after adding the dropout layer. Compared with Figure 6a, it is found that, compared with the regularization penalty, adding the dropout layer weakens the risk of overfitting to a certain extent.

### 4.4. Comparison Experiments

**Figure 9:**
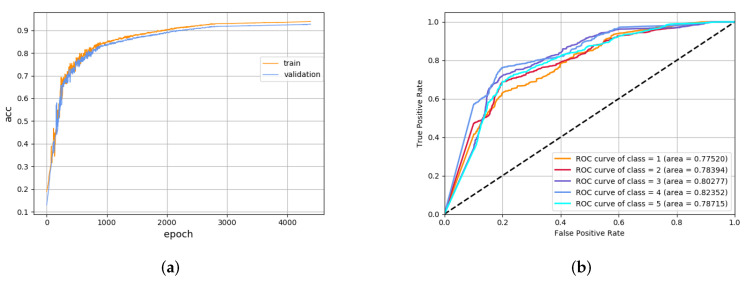
Model accuracy and ROC curve after optimization. (**a**) Accuracy of the optimized model. (**b**) ROC curve of the optimized model.

We have constructed the HCW ensemble model optimized by the dropout layer through the above experiments. The performance of the optimized model on the dataset is shown in Figure 9. Compared with the initial experimental results, the classification accuracy and the AUC value have improved. The comparison results can be seen in Table 4, comparing the complexity of several models and their accuracy on the test set, composed of mainly three types. The first is the classic time series data series prediction model, including KShape and LSTM. The second is a hybrid model that combines classic models, including KNN-DTW hybrid model with KL divergence measurement and the Gaussian mixture model (GMM). The last one is the partial HMM model, CWAE generation model, and HCM ensemble model after fusion optimization.

The optimized HCW model has higher accuracy than before optimization, which indicates that the dropout layer effectively prevents overfitting. The accuracy of the ensemble model is more effective than the classifier alone, which validates the previous analysis of the effectiveness of the ensemble strategy. Meanwhile, compared with the classical RNN models LSTM and GRU, the optimized HCW is slightly more accurate. Furthermore, the number of parameters of HCW model is much smaller than that of LSTM, because LSTM model has three gates and two states. In contrast, the HMM model in HCW has a simple structure with only one state and no gate, so the number of parameters is much lower than that of LSTM. Similarly, GRU has only two gates and one state compared to LSTM, and the structure of GRU is relatively simple and requires fewer parameters for training. Therefore, compared with LightGBM based on other ensemble strategies, the stacking ensemble strategy can perform the classification task better.

## 5. Conclusions

The main purpose of this work is to analyze the characteristics of existing supervised learning algorithms in detail, use hidden variables to extract features of time series data, and design an HCW ensemble model based on HMM mining continuous information and CVAEWD mining discrete information, which solves shortcomings of existing time series data mining algorithms that only rely on time series data continuity information for analysis. The HCM ensemble model improves the accuracy and efficiency of time series analysis. At the same time, when using CVAEWD to extract data distribution characteristics, Wasserstein distance is used instead of KL divergence. W-distance can better measure the similarity characteristics of two sequence distributions and has stronger generalization than KL divergence. Due to the characteristics of ensemble learning, the HCM model is more conducive to parallelism and can improve the operating efficiency.

## Figures and Tables

**Table 1:** LSH threshold and save data ratio information.

Class	1	2	3	4	5
Threshold	0.20	0.29	0.20	0.28	0.29
Save	90.76%	90.05%	94.06%	90.18%	94.61%

**Table 2:** Weight distribution in the fusion of the weighted method.

Weight	Class				
	1	2	3	4	5
α	0.392	0.566	0.492	0.503	0.431
β	0.608	0.434	0.508	0.497	0.569

**Table 3:** CVAEWD hidden parameter variable classifier parameters and structure.

Layer (Type)	Output Shape	Param
Linear	[64, 80]	5200
ReLU	[64, 80]	0
Dropout	[64, 80]	0
Linear	[64, 20]	1620
ReLU	[64, 20]	0
Dropout	[64, 20]	0
Linear	[64, 5]	105

**Table 4:** Comparison of the results of different algorithms.

Algorithms	Parameter	Highest Accuracy
KShpae	—	91.652%
LSTM	22,548,033	92.237%
GRU	13,765,473	91.714%
KNN-DTW	—	86.851%
GMM	—	83.830%
HMM	—	84.397%
CVAEWD	3,790,753	86.386%
CVAE	3,790,753	84.071%
**HCW**	**3,790,753**	**91.052%**
**HCW (optimized)**	**3,895,968**	**92.714%**
LightGBM	3,790,753	88.415%
